# Deletion of SHP-2 in mesenchymal stem cells causes growth retardation, limb and chest deformity, and calvarial defects in mice

**DOI:** 10.1242/dmm.012849

**Published:** 2013-09-25

**Authors:** Philip E. Lapinski, Melissa F. Meyer, Gen-Sheng Feng, Nobuhiro Kamiya, Philip D. King

**Affiliations:** 1 Department of Microbiology and Immunology, University of Michigan Medical School, Ann Arbor, MI 48109, USA; 2 Department of Pathology, University of California San Diego, San Diego, CA 92093, USA; 3 Center for Excellence in Hip Disorders, Texas Scottish Rite Hospital for Children, Dallas, TX 75219, USA

## Abstract

In mice, induced global disruption of the *Ptpn11* gene, which encodes the SHP-2 tyrosine phosphatase, results in severe skeletal abnormalities. To understand the extent to which skeletal abnormalities can be attributed to perturbation of SHP-2 function in bone-forming osteoblasts and chondrocytes, we generated mice in which disruption of *Ptpn11* is restricted to mesenchymal stem cells (MSCs) and their progeny, which include both cell types. MSC-lineage-specific SHP-2 knockout (MSC SHP-2 KO) mice exhibited postnatal growth retardation, limb and chest deformity, and calvarial defects. These skeletal abnormalities were associated with an absence of mature osteoblasts and massive chondrodysplasia with a vast increase in the number of terminally differentiated hypertrophic chondrocytes in affected bones. Activation of mitogen activated protein kinases (MAPKs) and protein kinase B (PKB; also known as AKT) was impaired in bone-forming cells of MSC SHP-2 KO mice, which provides an explanation for the skeletal defects that developed. These findings reveal a cell-autonomous role for SHP-2 in bone-forming cells in mice in the regulation of skeletal development. The results add to our understanding of the pathophysiology of skeletal abnormalities observed in humans with germline mutations in the *PTPN11* gene (e.g. Noonan syndrome and LEOPARD syndrome).

## INTRODUCTION

Src homology-2 protein tyrosine phosphatase (SHP-2), encoded by the *PTPN11* gene in humans, is a ubiquitously expressed intracellular signaling molecule that contains a pair of N-terminal protein phosphotyrosine-binding SH2 domains and a C-terminal protein tyrosine phosphatase (PTP) domain (Chong and Maiese, 2007; Matozaki et al., 2009). SHP-2 functions downstream of numerous cell surface receptors to regulate diverse cellular responses, including growth, survival, proliferation and differentiation. One principal way in which SHP-2 is known to act is to promote the coupling of cell surface receptors to activation of the Ras small GTP-binding protein (Dance et al., 2008). In its activated state, Ras triggers the activation of ERK mitogen activated protein kinases (MAPKs) and phosphatidylinositol 3-kinase (PI3K)–protein-kinase-B (PKB/AKT) signaling pathways that regulate cell responses in part through the mobilization of transcription factors (Buday and Downward, 2008). In addition, SHP-2 can also function as a negative regulator of intracellular signal transduction. For example, SHP-2 has been shown to inhibit the activation of STAT transcription factors, the p38 MAPK kinase and the RhoA small GTP-binding protein (Kontaridis et al., 2004; Chong and Maiese, 2007; He et al., 2013).

Gene targeting by homologous recombination in embryonic stem cells affords the opportunity to understand gene function in different physiological systems in the context of the whole animal. Mice that are homozygous for a non-conditional null allele of *Ptpn11* die at an early point in embryogenesis as a consequence of defective gastrulation (Saxton et al., 1997). Therefore, to understand the function of SHP-2 in tissue homeostasis in adults, we previously reported upon the generation and analysis of inducible SHP-2-deficient mice that are homozygous for a floxed allele of *Ptpn11* (*Ptpn11^fl/fl^*) and carry an *Ert2cre* transgene under control of the ubiquitin promoter (*Ub-Ert2cre*) (Bauler et al., 2011). Administration of tamoxifen to adult *Ptpn11^fl/fl^Ub-Ert2cre* mice resulted in *de novo* disruption of the *Ptpn11* gene and loss of SHP-2 protein in all tissues. Mice developed disorders of hematopoiesis, acanthosis of skin and weight loss, and the vast majority of mice died 6–8 weeks after gene deletion. In addition, mice developed severe skeletal malformations that included scoliosis (lateral curvature) and kyphosis (dorsal curvature) of the spine (Bauler et al., 2011). Cartilaginous growth plates of long bones and vertebrae were disorganized, and all bones of mice showed evidence of osteopetrosis (increased density). Osteopetrosis was attributed to impaired generation of osteoclasts, which are responsible for bone resorption and maintenance of bone homeostasis. Defective osteoclastogenesis in these mice was shown to be intrinsic to the osteoclast lineage and was caused by impaired macrophage colony stimulating factor receptor (M-CSFR) signal transduction in bone marrow osteoclast precursors, which is essential for their survival and expansion (Asagiri and Takayanagi, 2007; Bauler et al., 2011).

**TRANSLATIONAL IMPACT**

**Clinical issue**

Missense mutations in the *PTPN11* gene, which encodes the SHP-2 protein tyrosine phosphatase, are responsible for 50% of cases of Noonan syndrome and 95% of cases of LEOPARD syndrome in humans. These syndromes have several overlapping features that include growth retardation and other skeletal abnormalities such as chest malformation. Inactivating mutations of *PTPN11* have also been implicated in cases of metachondromatosis, which is characterized by the development of benign cartilaginous tumors. Understanding the precise role of SHP-2 in skeletal development is the first step towards elucidating the pathophysiology of skeletal abnormalities, which could reveal therapeutic candidates for treating the diverse spectrum of disorders associated with *PTPN11* mutations.

**Results**

In an earlier study, the authors used an inducible murine model of SHP-2 deficiency to demonstrate a crucial role for SHP-2 in the development of osteoclasts – a type of bone cell that is involved in bone resorption. Here, the authors disrupted the *Ptpn11* gene specifically in mesenchymal stem cells (MSCs) – the precursors of both osteoblasts and chondrocytes (cells found in cartilage) – to investigate whether the role of SHP-2 in bone development is restricted to specific cells. MSC-specific SHP-2 knockout mice showed several skeletal abnormalities that included postnatal growth retardation, limb and chest skeletal malformation, and calvarial defects. These abnormalities, which are not observed in mice with SHP-2 deficiency induced in adulthood, were shown to be associated with blocked differentiation of osteoblasts and dysregulated development of chondrocytes. The authors provide evidence that maturation of these cell lineages is disrupted as a consequence of defective activation of mitogen activated protein kinases and the AKT kinase.

**Implications and future directions**

This work identifies SHP-2 as an important cell-autonomous regulator of osteoblast and chondrocyte differentiation in mice. In light of the dramatic phenotypes observed in mice, altered activity of SHP-2 within MSC-lineage cells is likely to be a major contributing factor to the development of skeletal abnormalities in humans with inherited *PTPN11* mutations. Key cellular signaling pathways are shown to be involved, which could be of therapeutic relevance. In addition, the study demonstrates that the new mouse model described can be used as a powerful tool for further dissecting the role of SHP-2 in mammalian bone development.

Germline mutations of the *PTPN11* gene also cause skeletal abnormalities in three distinct autosomal-dominant human diseases. Missense mutations of *PTPN11* are the cause of 50% of cases of Noonan syndrome (NS; OMIM 163950) and 95% of cases of LEOPARD syndrome (LS; OMIM 151100) (Tartaglia et al., 2001; Digilio et al., 2002; Legius et al., 2002; Tartaglia et al., 2002; Zenker et al., 2004; Jongmans et al., 2005; Tartaglia et al., 2010; Martinez-Quintana and Rodriguez-González, 2012). These syndromes share a number of common features, including facial dysmorphism, cardiovascular and neurological abnormalities, hearing loss, and growth retardation (Tartaglia et al., 2010; Martinez-Quintana and Rodriguez-González, 2012). Apart from growth defects, other skeletal abnormalities that occur in these syndromes are scoliosis and kyphosis, pectus excavatum (concave chest) and pectus carinatum (convex chest) (Ho et al., 1989; Lee et al., 2001; Sarkozy et al., 2004; Noonan, 2006).

In resting unstimulated cells, SHP-2 exists mostly in an inactive conformation as a result of physical interaction between the most N-terminal SH2 domain and the PTP domain; this interaction blocks access of the PTP domain to substrates (Barford and Neel, 1998; Hof et al., 1998). In stimulated cells, recognition of phosphotyrosyl ligands within receptor tyrosine kinases or adapter proteins by the N-terminal SH2 domain causes a conformational change of the SH2 domain that results in release from the PTP domain and enzyme activation. Most NS *PTPN11* mutations disrupt this inhibitory intramolecular interaction (Fragale et al., 2004; Keilhack et al., 2005; Tartaglia et al., 2006; Martinelli et al., 2008; Tartaglia et al., 2010). Consequently, these mutations result in increased basal and stimulated PTP activity. By contrast, in LS, the most common mutations result in reduced catalytic activity (Hanna et al., 2006; Kontaridis et al., 2006; Tartaglia et al., 2006). However, as evidenced by the fact that *Ptpn11* hemizygous mice are normal, LS is not simply a consequence of haploinsufficiency and how LS *PTPN11* mutations cause disease is not completely understood (Arrandale et al., 1996). Evidence has been put forth in favor of dominant-negative behavior of *PTPN11* mutations in LS (Kontaridis et al., 2006; Marin et al., 2011). In addition, it has been shown that LS mutations promote the open conformation of SHP-2 (Kontaridis et al., 2006; Yu et al., 2013), which might compensate for the direct affect of mutations upon catalytic activity, resulting in a gain of function similar to NS (Yu et al., 2013). Interestingly, as evidenced in a knock-in mouse model of LS, as well as in cell lines derived from individuals with LS, LS mutations seem to result in blocked activation of MAPK but increased activation of AKT downstream of receptor tyrosine kinases (Edouard et al., 2010; Marin et al., 2011). The increased AKT activation might result from decreased dephosphorylation of adaptor proteins that interact with PI3K, resulting in increased PI3K activation in the respective signaling pathways.

Recently, germline mutations of *PTPN11* have also been identified in approximately 50% of cases of another bone disease, metachondromatosis (OMIM 156250), which is characterized by the development of benign exostotic and endosteal cartilagenous tumors (Kennedy, 1983; Bowen et al., 2011). *PTPN11* mutations in metachondromatosis include frame-shift, nonsense and splice-site mutations that are considered inactivating. The disease is thought to depend upon acquisition of somatic second-hit mutations in the wild-type *PTPN11* allele, with resulting complete loss of SHP-2 function in affected cells (Bowen et al., 2011).

Given the importance of SHP-2 in the regulation of bone phenotype in humans, in the current study we investigated whether SHP-2 has a cell-autonomous function in the chondrocyte lineage and also whether it regulates the development and function of osteoblasts. For this purpose, we generated mice with a targeted deletion of SHP-2 in mesenchymal stem cells (MSCs), which are the precursors of both cell types. Interestingly, these mice showed severe abnormalities of chondrocyte and osteoblast differentiation and developed multiple skeletal defects that are not observed in the adult induced SHP-2-deficient model, including growth retardation, limb and chest skeletal abnormalities, and calvarial defects.

## RESULTS

### Growth retardation and early lethality in MSC SHP-2-deficient mice

**Fig. 1.**
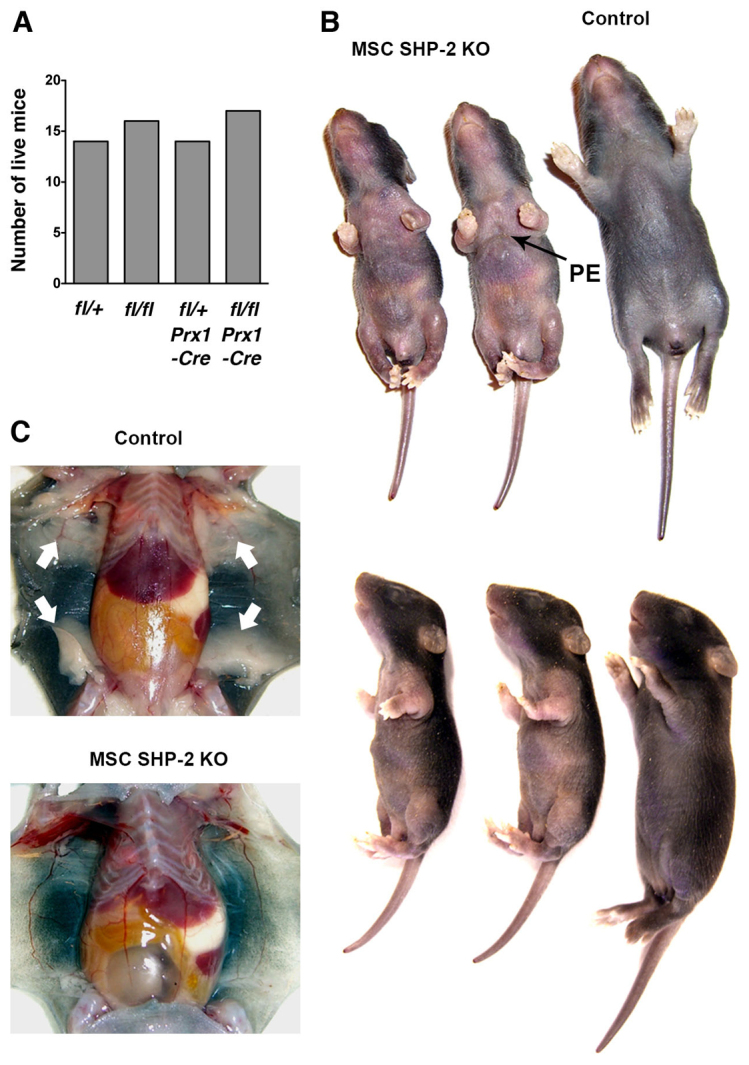
**Skeletal abnormalities in MSC SHP-2 KO mice.** (A) Number of pups of the indicated genotypes from crosses of *Ptpn11^fl/fl^* and *Ptpn11^fl/+^ Prx1-Cre* parents determined 8 days after birth (*n*=61 mice total). (B) Gross appearance of *Ptpn11^fl/fl^ Prx1-Cre* (MSC SHP-2 KO) pups compared with a littermate *Ptpn11^fl/+^ Prx1-Cre* pup (Control) at 8 days. Note overall reduced size and deformed limbs of MSC SHP-2 KO pups. Note also pectus excavatum (PE) in one of the MSC SHP-2 KO pups (indicated with arrow). (C) Shown are subcutaneous fat pads in a control and littermate MSC SHP-2 KO pup at 8 days (arrows). Note the absence of fat pads in the MSC SHP-2 KO pup. The same phenotypes shown in B and C were observed in all of greater than 40 examined MSC SHP-2 KO pups.

To examine the effect of SHP-2 deletion in MSC-lineage cells, we generated *Ptpn11^fl/fl^* mice, which carry a *Cre* transgene under control of the paired-related homeobox gene-1 (*Prx1*) promoter (*Ptpn11^fl/fl^Prx1-Cre* mice). The *Prx1* promoter in these mice directs expression of Cre to MSCs at least in limbs and head during development (Logan et al., 2002). In crosses of parental *Ptpn11^fl/fl^* and *Ptpn11^fl/+^Prx1-Cre* mice, the frequency of *Ptpn11^fl/fl^Prx1-Cre* pups was similar to that of *Ptpn11^fl/+^*, *Ptpn11^fl/fl^* and *Ptpn11^fl/+^Prx1-Cre* pups assessed 8 days after birth (Fig. 1A). Therefore, loss of SHP-2 expression in MSCs does not result in embryonic lethality. However, *Ptpn11^fl/fl^ Prx1-Cre* mice (hereafter referred to as MSC SHP-2 KO mice) were phenotypically abnormal. Although of similar size at birth, all MSC SHP-2 KO mice showed reduced growth and were visibly smaller than littermates by 2–3 days postpartum (Fig. 1B). In addition, MSC SHP-2 KO mice exhibited other skeletal abnormalities that included shortened and malformed limbs, and pectus excavatum (concave chest) or pectus carinatum (convex chest) (Fig. 1B). The same skeletal and growth abnormalities were observed in female and male mice.

MSC SHP-2 KO mice did not survive beyond 3 weeks of age. Notably, on postmortem examination, all MSC SHP-2 KO mice lacked subcutaneous fat (Fig. 1C), which is consistent with the MSC origin of adipocytes and the recent finding that SHP-2 is essential for adipogenesis (He et al., 2013). In adipocyte-specific SHP-2-deficient mice, it has been established that lipodystrophy (loss or absence of fat) is the cause of premature death (He et al., 2013). Therefore, absence of subcutaneous fat is probably one cause of death of MSC SHP-2 KO mice. In addition, reduced suckling as a consequence of impaired mobility might contribute to premature lethality. *Ptpn11^fl/+^ Prx1-Cre* pups were phenotypically indistinguishable from *Ptpn11^fl/+^* and *Ptpn11^fl/fl^* mice, and were used as controls in subsequent experiments.

### Limb and chest abnormalities in MSC SHP-2 KO mice

**Fig. 2.**
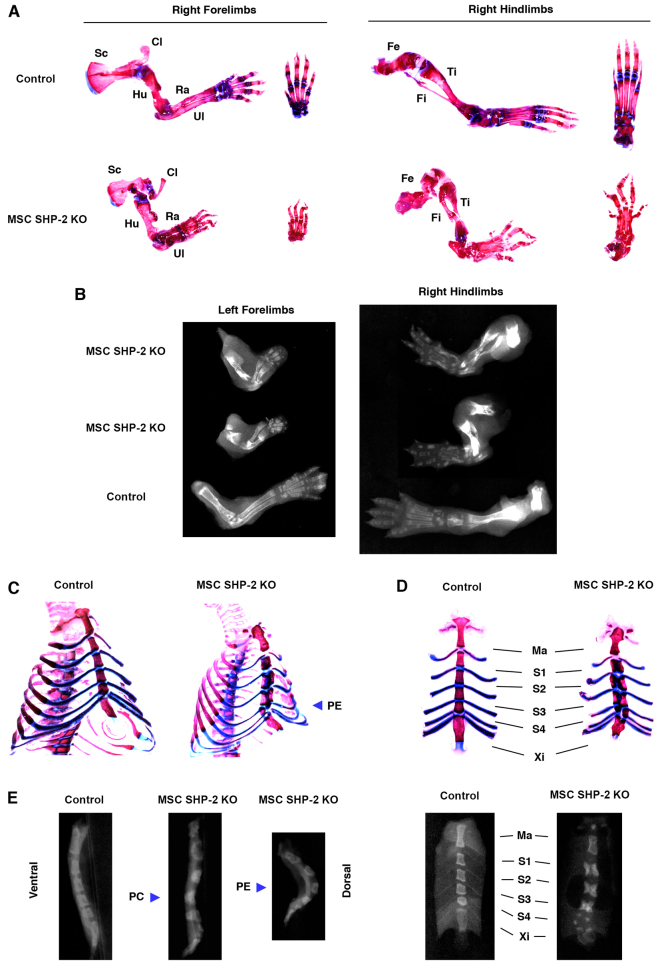
**Limb deformity and chest abnormalities in MSC SHP-2 KO mice.** (A) Right forelimbs and hindlimbs of an MSC SHP-2 KO and littermate control mouse at 11 days of age stained with alizarin red and alcian blue. Additional images of hands and feet are shown to the right of the respective images. Sc, scapula; Cl, clavicle; Hu, humerus; Ra, radius; Ul, ulna; Fe, femur; Ti, tibia; Fi, fibula. (B) X-rays of left forelimbs and right hindlimbs of MSC SHP-2 KO and littermate control mice. Analyses were performed on 8-day-old mice. Note shortened, disorganized and malformed limb bones in MSC SHP-2 KO mice. (C) Whole chest mounts from MSC SHP-2 KO and control littermate mice at 11 days of age stained with alizarin red and alcian blue. Note pectus excavatum (PE) in the MSC SHP-2 KO mouse. (D) Isolated sternums from the mice shown in C. Ma, manubrium; S1–S4, sternebrae 1–4; Xi, xiphoid process. Note ectopic cartilage in S1–S4 of MSC SHP-2 KO mice that separates bone. (E) X-ray images of sternums of MSC SHP-2 KO and littermate control mice at 8 days. Lateral images are grouped at left. Note pectus carinatum (PC) in one MSC SHP-2 KO mouse and pectus excavatum (PE) in another. Ventral X-ray images are shown at right. Note the appearance of S1–S3 as pairs of bones and S4 and the xiphoid process as multiple bone fragments in the MSC SHP-2 KO mouse.

To characterize the skeletal abnormalities of MSC SHP-2 KO mice further, pups were euthanized at 1–2 weeks of age and fixed skeletal whole mounts were stained with alizarin red and alcian blue to directly visualize bone and cartilage, respectively. In addition, X-ray images of skeletons were taken. Both types of analysis confirmed the severe limb deformity in SHP-2-deleted mice (Fig. 2A,B). Scapulae, clavicles, humeri, radii, ulnae and all hand bones were reduced in size and malformed. Similarly, femora, tibiae, fibulae and all foot bones were highly misshapen.

Pectus excavatum and pectus carinatum were readily apparent in chest skeletal whole mounts stained with alizarin red/alcian blue (Fig. 2C,D) and in lateral X-ray images of sternae of MSC SHP-2 KO mice (Fig. 2E). Approximately 80% of MSC SHP-2 KO pups showed pectus excavatum and the remaining 20% showed pectus carinatum. Regardless of which type of chest deformity was present, alizarin red/alcian blue staining and ventral X-ray images (Fig. 2E) of sternae revealed abnormal ossification of the sternum. The manubrium of MSC SHP-2 KO mice, although misshapen, was intact. However, sternebrae 1–3 presented as pairs of bones separated at the midline. Furthermore, sternebrum 4 contained at least three bone fragments and the xiphoid process was also abnormal, consisting of unfused or partially fused pairs of bones (Fig. 2C–E). Ectopic cartilage was identified at the midline between the pairs of bones within sternebrae 1 through 3 and surrounding bone fragments within sternebrum 4 (Fig. 2C,D).

Pelvic bones were also shortened and deformed in MSC SHP-2 KO mice (data not shown). However, all other non-calvarial bones (see below), although reduced in size, were normal in shape.

### Calvarial defects in MSC SHP-2 KO mice

**Fig. 3.**
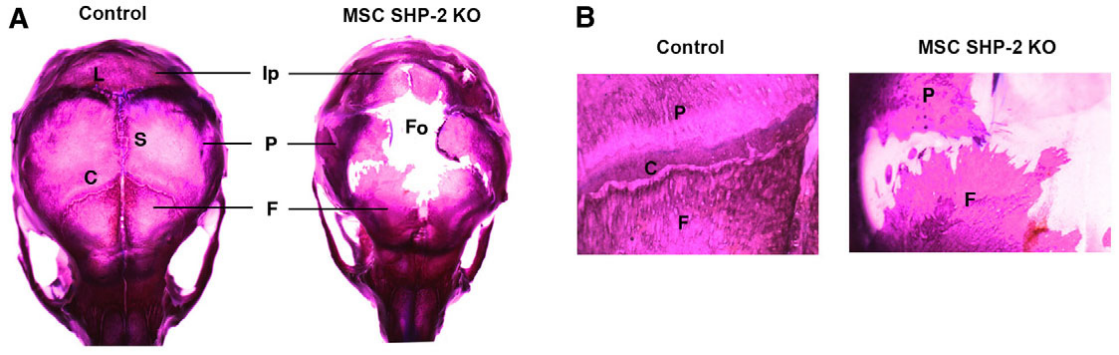
**Impaired calvarial development in MSC SHP-2 KO mice.** (A) Calvaria from an 11-day-old MSC SHP-2 KO mouse and a littermate control stained with alizarin red and alcian blue. Ip, interparietal bone; P, parietal bone; F, frontal bone; L, lambdoid suture; S, sagittal suture; C, coronal suture; Fo, fontanelle. Note impaired growth of interparietal, parietal and frontal bones and large fontanelle resulting from failure of bone fusion in MSC SHP-2 KO mice. (B) Higher power images of skulls in A, showing region of coronal suture.

Distinct from long bones and sternae, which develop by endochondral ossification, the calvarial bones develop by intramembranous ossification. Defects in the development of calvarial bones were also noted in MSC SHP-2 KO mice (Fig. 3A,B). In control mice at 11 days of age, all frontal, parietal and interparietal bones had formed and sutures between bones had developed. By contrast, in MSC SHP-2 KO mice at the same age, none of these skull bones had formed properly and fusion had not initiated or was incomplete (Fig. 3A,B). Consequently, the skulls of MSC SHP-2 KO mice contained a large centrally located fontanelle.

### Histological analysis of limbs and sternae of MSC SHP-2 KO mice

**Fig. 4.**
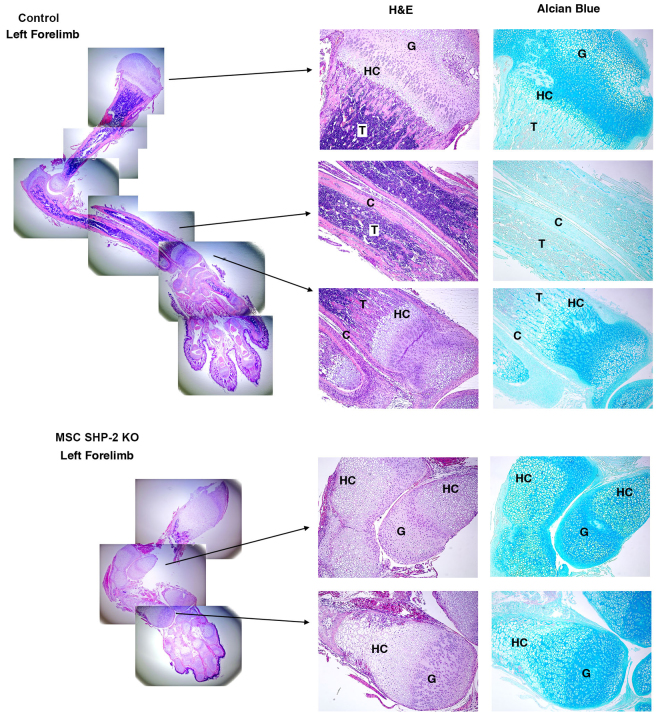
**Histology of MSC SHP-2 KO forelimbs.** At left are shown composites of low-power images of H&E-stained sections of whole forelimbs of MSC SHP-2 KO and control littermate mice at 12 days (40×). At right are shown higher power images of the indicated select regions stained with H&E and of adjacent serial sections stained with alcian blue (100×). G, growth plate; HC, hypertrophic chondrocyte region; C, cortical bone; T, trabecular bone. Features to note include the near absence of cortical bone and massively extended hypertrophic chondrocyte region in MSC SHP-2 KO mice.

Histological analyses were next performed to understand the cellular basis of limb and chest skeletal abnormalities in MSC SHP-2 KO mice. Shown in Fig. 4 are the results of analyses performed upon entire left forelimbs. The same results were obtained with other limbs of MSC SHP-2 KO mice. Ossification of long bones and hand bones was highly abnormal. Cortical bone was essentially absent and the amount of trabecular bone was also much reduced in MSC SHP-2 KO mice compared with controls. Chondrocytogenesis was also highly abnormal in long bones and hands. Cartilagenous growth plates were disorganized and a normal columnar morphology of differentiating chondrocytes was not observed in the MSC SHP-2 KO mice. Most strikingly, the region of hypertrophic chondrocytes was massively extended in MSC SHP-2 KO mice and, in long bones, hypertrophic chondrocytes were the major cell type (Fig. 4).

In sternae of MSC SHP-2 KO mice, in the manubrium and all sternebrae, cortical bone was absent and the amount of trabecular bone was much reduced compared with controls (supplementary material Fig. S1). In addition, cartilaginous growth plates were disorganized in sternal bones and the region of hypertrophic chondrocytes was greatly extended, particularly in sternebrae (supplementary material Fig. S1). Thus, the same pathological process seems to underlie limb and chest skeletal abnormalities in MSC SHP-2 KO mice.

### Molecular analysis of osteoblast and chondrocyte differentiation in MSC SHP-2 KO mice

**Fig. 5.**
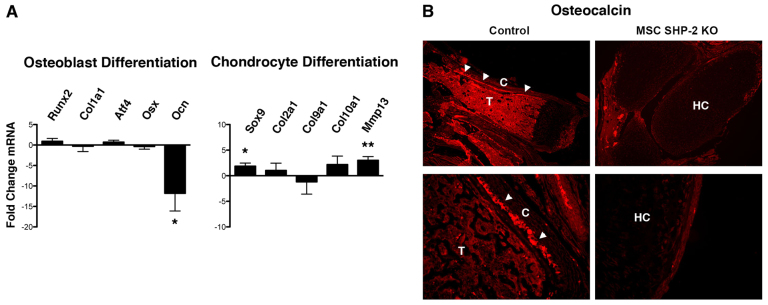
**Osteogenic and chondrocytic differentiation in MSC SHP-2 KO mice.** (A) Relative expression of the indicated osteoblast- and chondrocyte-lineage markers in long bones of hindlimbs from pairs of MSC SHP-2 KO and littermate control mice (age range 6–16 days) was determined by real-time quantitative PCR. Runx2, runt-related transcription factor 2; Col1a1, collagen type 1 alpha 1; Atf4, activating transcription factor 4; Osx, osterix; Ocn, osteocalcin; Sox9, SRY (sex-determining region Y)-box 9 protein; Col2a1, collagen type 2 alpha 1; Col9a1, collagen type 9 alpha 1; Col10a1, collagen type 10 alpha 1; Mmp13, matrix metallopeptidase 13. A fold change in mRNA expression in MSC SHP-2 KO mice compared with control mice was determined (positive value for increase, negative value for decrease). Experiments were performed with eight littermate pairs of mice. Shown is the mean plus 1 s.e.m. of the fold change in mRNA expression. The statistical significance of fold changes (from an expected value of zero under the null hypothesis of no change) was determined using a Student’s one-sample *t*-test. *P<0.05, **P<0.01. (B) Sections of forelimbs of MSC SHP-2 KO and littermate control mice were stained with an anti-osteocalcin antibody. Low-power images (100×) are shown at top and higher-power images (400×) are shown at bottom. C, cortical bone; T, trabecular bone; HC, hypertrophic chondrocyte region. Note mature osteoblasts (arrowheads) adjacent to cortical bone in control mice and absence in SHP-2-deleted mice.

To understand the mechanism of impaired osteogenesis and altered chondrocytogenesis in MSC SHP-2 KO mice, we examined expression of osteoblast and chondrocyte differentiation markers in long bones by real-time quantitative PCR (Fig. 5A). With regards to osteoblast differentiation, expression of the early osteoblast marker *Col1a1* was not different between MSC SHP-2 KO mice and controls. Similarly, expressions of *Runx2*, *Osterix* and *Atf4*, which function as master transcription factors of the osteoblast lineage, were normal. In contrast, expression of osteocalcin, a marker of mature osteoblasts, was reduced more than tenfold (Fig. 5A). Therefore, in MSC SHP-2 KO mice, osteoblast differentiation is blocked at a terminal stage, downstream of expression of master transcription factors.

To confirm loss of osteocalcin expression and mature osteoblasts in MSC SHP-2 KO mice, we performed immunohistochemical staining of long bones using an anti-osteocalcin antibody (Fig. 5B). Osteoblasts expressing osteocalcin were readily identified in bone sections of control mice, particularly adjacent to regions of cortical bone. However, osteocalcin protein was not detected in long bones of MSC SHP-2 KO mice.

With regards to chondrocytes, expression of the master transcription factor of chondrogenic differentiation, *Sox9*, was significantly increased in MSC SHP-2 KO mice compared with controls (Fig. 5A). However, expression of the chondrocyte markers *Col2a1* and *Col9a1* was not significantly changed compared with control mice. Likewise, expression of *Col10a1*, a marker of hypertrophic chondrocytes, was unchanged. In contrast, expression of *Mmp13*, a marker of terminally differentiated hypertrophic chondrocytes, was significantly increased in MSC SHP-2 KO mice compared with controls (Fig. 5A). This finding, which is consistent with histological findings, indicates that MSC SHP-2 KO mice accumulate large numbers of terminally differentiated hypertrophic chondrocytes.

### Impaired MAPK and AKT activation in bones of MSC SHP-2 KO mice

**Fig. 6.**
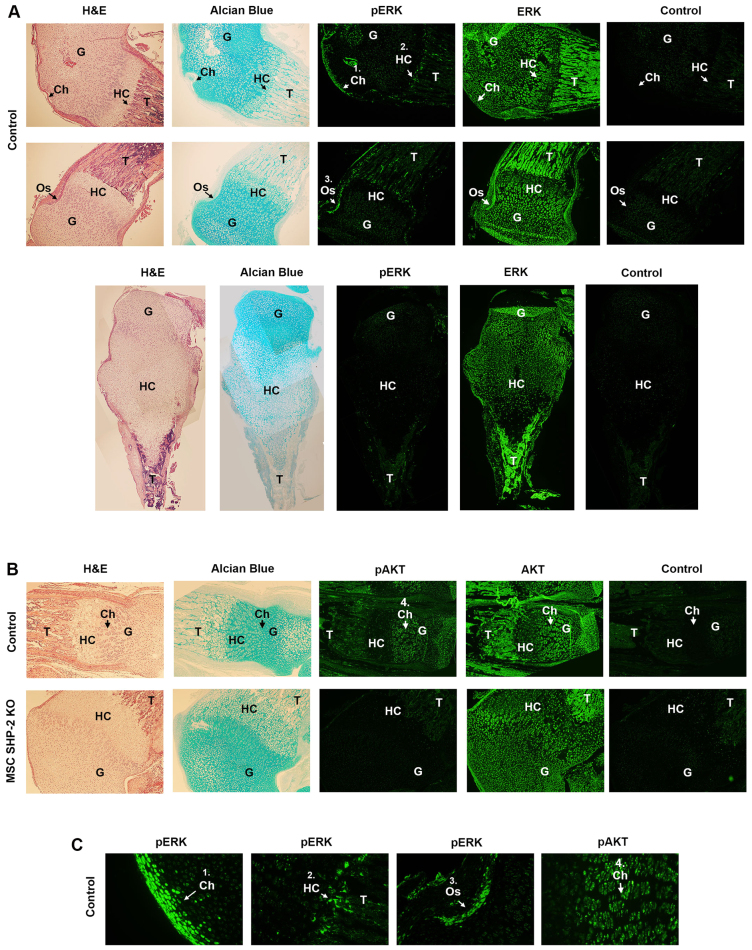
**Loss of ERK and AKT activation in bone-forming cells of MSC SHP-2 KO mice.** (A) Serial sections of left hindlimbs of MSC SHP-2 KO and littermate control mice at 12 days of age were stained with H&E, alcian blue, anti-phospho-ERK (pERK) or total ERK antibodies followed by secondary reagents (green) or with secondary detection reagents alone (control). For control mice, two different sets of serial sections are shown (top two rows, 100×). For MSC SHP-2 KO mice, composite images of serial sections of an entire femur bone are depicted (100×). (B) Serial sections of left hindlimbs of MSC SHP-2 KO and control littermate mice at 12 days of age were stained with H&E, alcian blue, anti-phospho-AKT (pAKT) or total AKT antibodies followed by secondary reagents (green) or with secondary detection reagents alone (control). All images, 100×. (C) Shown are higher-power images of the numbered regions (1–4) from A and B (400×). G, growth plate; T, trabecular bone; Ch, chondrocytes; HC, hypertrophic chondrocytes; Os, osteoblasts. Note absence of pERK and pAKT staining in MSC SHP-2 KO mice.

SHP-2 promotes the activation of the Ras pathway downstream of growth factor receptors in numerous cell types (Dance et al., 2008; Matozaki et al., 2009). In turn, Ras triggers the activation of downstream MAPK and PI3K-AKT effector pathways. Therefore, we investigated whether activation of MAPK and AKT was inhibited in bone-forming cells in MSC SHP-2 KO mice (Fig. 6). For this purpose, we performed immunohistochemical staining of limbs using phospho-specific anti-MAPK and -AKT antibodies that detect only the activated forms of these kinases. In control mice, activated MAPK was readily detected within osteoblasts, chondrocytes and hypertrophic chondrocytes, and activated AKT was readily detected in chondrocytes. By contrast, activated MAPK and AKT could not be detected in bone-forming cells of MSC SHP-2 KO mice. For comparison, we also examined activation of the LIMK1 kinase using a phospho-specific anti-LIMK1 antibody (supplementary material Fig. S2). LIMK1 lies downstream of the RhoA small GTP-binding protein, which might also be regulated by SHP-2 (Maekawa et al., 1999; Ohashi et al., 2000). In both control and MSC SHP-2 KO bones, activated LIMK1 was most apparent in chondrocytes. However, no obvious difference in the intensity of phospho-LIMK1 staining was detected between the two types of mice.

## DISCUSSION

The aim of the current studies was to examine the role played by SHP-2 within MSC-lineage cells in the regulation of skeletal phenotype. We demonstrate that loss of SHP-2 in the MSC lineage results in reduced postnatal growth, limb malformation, pectus excavatum and pectus carinatum. In addition, calvarial bone development was delayed.

Histological examination of limbs and sternae of MSC SHP-2 KO mice revealed a reduced bone density, particularly of cortical bone. Analysis of the expression of bone differentiation markers in MSC SHP-2 KO mice by quantitative PCR indicated that mature osteoblasts were much reduced in number and this was confirmed by immunohistochemical staining for osteocalcin. Conversely, adult induced SHP-2-deficient mice show a large increase in bone density (Bauler et al., 2011). Presumably, in adult induced SHP-2-deficient mice, osteopetrosis develops because the influence of osteoclast loss is more significant than the effect of any blocked osteoblast differentiation upon bone density. Interestingly, in individuals with NS, reduced bone mineral density has also been recorded when patients were matched to controls for age, gender, height and ethnicity (Choudhry et al., 2012).

Cartilage was highly abnormal in sternae and limbs of MSC SHP-2 KO mice. In sternebrae and the xiphoid process, ectopic cartilage separated bones into two or more fragments. Histological analysis revealed highly dysregulated chondrogenic differentiation with disordered chondrocyte columnar stacks and a large increase in the number of hypertrophic chondrocytes, which was the major cell type between pieces of bone. Similarly, chondrocytic differentiation was highly disorganized in long bones and bones of hands and feet, and was associated with a large increase in the number of hypertrophic chondrocytes. Quantitative PCR analysis of chondrocytic differentiation in long bones revealed a significant increase in the number of terminally differentiated hypertrophic chondrocytes. Abnormal chondrocytic differentiation and osteoblast differentiation is thus probably a major factor in the development of sternal and limb abnormalities in MSC SHP-2 KO mice. Dysregulated chondrocyte differentiation and an increase in the number of hypertrophic chondrocytes were also observed in long bones of adult induced SHP-2-deficient mice (Bauler et al., 2011), albeit that abnormalities of chondrocyte differentiation were mild in comparison with MSC SHP-2 KO mice. That the same chondrocyte phenotype is observed in MSC SHP-2 KO mice therefore shows that the role of SHP-2 as a regulator of chondrogenic differentiation is intrinsic to the MSC lineage. Of note, pectus excavatum, pectus carinatum and disruption of sternal bones are not apparent in adult induced SHP-2-deficient mice. This indicates that disruption of normal SHP-2 function throughout development is necessary for these sternal abnormalities to occur.

Activated ERK and AKT kinases could not be detected in bone-forming cells *in situ* in MSC SHP-2 KO mice. By contrast, activation of LIMK, which, at least in myogenic cells, lies downstream of RhoA and SHP-2, was unaffected in bone-forming cells of MSC SHP-2 KO mice. Notably, mice that lack expression of ERK1 and ERK2 specifically in MSC-lineage cells develop a skeletal phenotype that is strikingly similar to that described here in MSC SHP-2 KO mice (Matsushita et al., 2009). Thus, MSC ERK KO mice show severe limb deformity associated with lack of cortical bone, ectopic cartilage formation and a large increase in the number of hypertrophic chondrocytes. Furthermore, terminal osteoblast differentiation but not earlier stages of osteoblast differentiation were blocked in these mice and there was a marked increase in the number of terminally differentiated hypertrophic chondrocytes. Therefore, impaired ERK activation is likely to be a major factor that accounts for the development of skeletal abnormalities in MSC SHP-2 KO mice. In addition, impaired activation of AKT is likely to contribute to the phenotype. Thus, mice that lack AKT1 and AKT2 isoforms show dwarfism and delayed bone development associated with blocked osteogenic differentiation (Peng et al., 2003; Mukherjee et al., 2010). Conversely, the PI3K-AKT axis is known to inhibit terminal hypertrophic chondrocyte differentiation (Kita et al., 2008).

Postnatal growth retardation is a recognized feature of NS and LS. Approximately 70% of adults with NS are below the 10th percentile for height (Noonan et al., 2003) and 85% of adults with LS are below the 25th percentile (Gorlin et al., 1969). Similarly, *Ptpn11* knock-in mice that carry *PTPN11* mutations commonly found in NS or LS, i.e. D61G and Y279C, respectively, both show postnatal growth retardation (Araki et al., 2004; Marin et al., 2011). Although controversial, in NS, the consensus of opinion is that growth abnormalities are in part a consequence of decreased responsiveness to growth hormone (GH), as evidenced by reduced levels of insulin-like growth factor 1 (IGF-1) (Binder et al., 2005; Limal et al., 2006; Noordam et al., 2008; De Rocca Serra-Nédélec et al., 2012). In *Ptpn11* D61G NS mice, blockade of MAPK signaling restores IGF-1 levels and postnatal growth, consistent with the idea that reduced IGF-1 and growth retardation in these mice is dependent upon hyperactivation of ERK in the GH signaling pathway (De Rocca Serra-Nédélec et al., 2012). In these same studies, knockdown of SHP-2 in a fibroblast cell line was found not to influence GH-induced activation of AKT, although previous studies of SHP-2 null cells have shown clearly that SHP-2 is required for AKT activation downstream of other receptors such as platelet derived growth factor (PDGF) and the IGF-1 receptor (Hakak et al., 2000; Wu et al., 2001; De Rocca Serra-Nédélec et al., 2012). The molecular basis of growth defects in LS is less well understood. Cells from *Ptpn11* Y279C LS mice show blocked MAPK activation and increased AKT activation in response to IGF-1, which might relate to growth defects, although this has not been directly examined (Marin et al., 2011). In MSC SHP-2 KO mice, based upon the phenotypes of ERK- and AKT-deficient mice discussed above, growth retardation is expected to be a consequence of blocked activation of both kinases.

Pectus excavatum and pectus carinatum are also common features of NS and LS. These sternal abnormalities have been observed in up to 95% of individuals with NS and 50% of those with LS (Sharland et al., 1992; Digilio et al., 2006). Pectus excavatum and carinatum have also been observed in *Ptpn11* Y279C LS mice (Marin et al., 2011). However, whether SHP-2 has an intrinsic role in bone-forming lineages in the regulation of skeletal growth and sternal development in individuals with NS or LS, or in mouse models of these syndromes, is unknown. In this regard, the findings in this study of growth defects and sternal abnormalities in MSC SHP-2 KO mice point to a crucial role for SHP-2 within bone-forming lineages for normal growth and chest development, and are consistent with the view that altered SHP-2 function within bone-forming cell lineages is at least partially responsible for both types of skeletal abnormality in NS and LS. In addition, the fact that similar phenotypes can result from gain of function, altered function or loss of function of SHP-2 in humans and mice indicates that a restricted range of SHP-2 activity is necessary for normal skeletal development.

Spinal curvature was not observed in the majority of MSC SHP-2 KO mice, although is common in individuals with NS and is also found in LS (Ho et al., 1989; Lee et al., 2001; Sarkozy et al., 2004; Noonan, 2006). This could indicate that spinal curvature in NS and LS, and in adult induced SHP-2-deficient mice, is dependent upon altered SHP-2 activity in other cell lineages or requires altered SHP-2 activity in other cell lineages in addition to osteoblasts and chondrocytes. However, spinal curvature is not observed in *Ptpn11* D61G NS or *Ptpn11* Y279C LS models either. For MSC SHP-2 KO mice, it might also be relevant that the *Prx1-Cre* transgene is expressed mostly in early limb bud and craniofacial mesenchyme starting at E9.5 of development (Logan et al., 2002). Interestingly, we have recently found that, in inducible chondrocyte-specific SHP-2-deficient mice, scoliosis does develop when deletion is initiated at the juvenile stage (Kim et al., 2013a). Potentially, therefore, spinal abnormalities resulting from altered SHP-2 activity might not manifest until adulthood and would not be observed in MSC SHP-2 KO mice because all mice die by 3 weeks of age. This possibility is consistent with the fact that spinal abnormalities in LS are observed most commonly in older individuals (Ho et al., 1989; Sarkozy et al., 2004).

Classical features of metachondromatosis were also not observed in MSC SHP-2 mice. In contrast, metachondromatosis was recently reported in *Ptpn11* conditional mice in which a cathepsin K (*Ctsk*)-promoter-driven *Cre* was used to disrupt *Ptpn11* in *Ctsk*-expressing cells, which included a subpopulation of MSCs (Yang et al., 2013). In addition, we have recently observed metachondromatosis in inducible chondrocyte-specific SHP-2-deficient mice (Kim et al., 2013b). However, in both of these models, features of metachondromatosis were not noted until adulthood. Therefore, metachondromatosis might not be observed in MSC SHP-2 KO mice again because of the early lethality.

Limb malformation was a prominent feature of MSC SHP-2 KO mice, although is not observed in humans with *PTPN11* mutations nor in *Ptpn11* D61G NS or *Ptpn11* Y279C LS mice. This difference can most likely be explained by the different nature of mutations. Thus, in individuals with NS and in *Ptpn11* D61G NS mice, mutations result in increased ERK activation; in individuals with LS and in *Ptpn11* Y279C LS mice, mutations results in loss of ERK activation and an increase in AKT activation; and in MSC SHP-2 KO mice, activation of both ERK and AKT is blocked. The reason for the difference between the effect of mutations in individuals with LS and *Ptpn11* Y279C LS mice compared with MSC SHP-2 KO mice is uncertain but probably relates to the fact that mutations in the former two cases do not result in complete loss of SHP-2 PTP activity, whereas, in MSC SHP-2 KO mice, SHP-2 PTP activity is absent.

With regards to the inactivating germline mutations in metachondromatosis, presumably these do not result in limb deformity because the inherited wild-type allele is sufficient for normal development. Furthermore, somatic second-hit mutations of the wild-type allele in individuals with metachondromatosis would predictably affect only a small percentage of cells and not impact upon limb development. In this regard, it is of note that, in chimeric mice that are generated from fusion of SHP-2 null and wild-type morulas, limb development proceeds normally (Saxton et al., 2000). However, consistent with the findings reported herein, SHP-2 null cells were excluded from limb buds of these chimeric mice, indicating a role for SHP-2 in limb development. Further analysis revealed a required role for SHP-2 in mesenchyme of the progress zone in limb buds. A similar function for the fibroblast growth factor receptor 1 (FGFR1) was noted in parallel morula aggregation experiments, suggesting a role for SHP-2 in the FGFR1 signaling pathway in the regulation of limb morphogenesis (Saxton et al., 2000).

In conclusion, we show here that specific deletion of SHP-2 in MSC-lineage cells results in several skeletal abnormalities, including postnatal growth retardation, limb deformity, pectus excavatum and pectus carinatum, and calvarial defects. At the cellular level, these abnormalities are associated with impaired osteoblast differentiation and a large increase in the number of terminally differentiated hypertrophic chondrocytes. At the molecular level, abnormalities reflect a required role for SHP-2 in the activation of ERK and AKT kinases in bone-forming cells. Findings illuminate further upon the role of SHP-2 as a regulator of bone and are pertinent to an understanding of the etiology of skeletal malformations in humans with *PTPN11* mutations.

## MATERIALS AND METHODS

### Mice

*Ptpn11^fl/fl^* mice have been described previously (Zhang et al., 2004). *Prx1-cre* mice were purchased from JAX (Logan et al., 2002). Mice were intercrossed to generate *Ptpn11^fl/+^ Prx1-cre* mice that were then backcrossed with *Ptpn11^fl/fl^* mice. Genotypes were determined by PCR of tail genomic DNA. All experiments were performed in compliance with University of Michigan guidelines and were approved by the University Committee on the Use and Care of Animals.

### X-ray imaging

Mice were fixed in 10% buffered formalin overnight. Limbs and sternae were carefully detached and X-ray images were acquired using a microradiography machine (Faxitron Corporation). Exposure was for 6 seconds at 25 kV with 4× magnification.

### Alizarin red and alcian blue staining

As much soft tissue as possible was removed from skeletons of deceased mice before fixation overnight in 95% ethanol. Skeletons were then stained overnight in a 0.5 mg/ml solution of alcian blue in 80% ethanol and 20% acetic acid. After two 3-hour washes in 95% ethanol, skeletons were treated with 1.5% KOH for 2 hours followed by overnight staining in a 0.15 mg/ml solution of alizarin red in 0.5% KOH. Skeletons were washed twice for 3 hours each in 1% KOH/20% glycerol before images were acquired on a dissecting microscope equipped with a digital camera (Nikon).

### Histology

Limbs and sternae from deceased mice were fixed overnight in formalin and were then decalcified by immersion in 10% EDTA-ammonium hydroxide, pH 7.2, for 2 weeks prior to paraffin embedding. Sections of tissues (5 μm) were stained with H&E or alcian blue and viewed on an upright microscope equipped with digital camera (Olympus).

### Immunohistology

For osteocalcin staining, sections of whole limbs were treated with HCl for 5 minutes, washed and stained with an anti-osteocalcin antibody (Santa Cruz Biotechnology) followed by secondary Alexa-Fluor-594-coupled anti-rabbit Ig (Jackson ImmunoResearch). For detection of activated kinases, sections were stained with anti-phospho-ERK (D13.14.4E, Cell Signaling), anti-phospho-AKT (193H12, Cell Signaling) and anti-phospho-LIMK1 (Biorbyt) antibodies followed by biotinylated anti-rabbit Ig (Jackson ImmunoResearch) and streptavidin-HRP with tyramide signal amplification (Perkin Elmer). For detection of total kinases, sections were stained with anti-ERK (Cell Signaling), anti-AKT (C67E7, Cell Signaling) and anti-LIMK1 (Abcam) antibodies, again followed by biotinylated anti-rabbit Ig and streptavidin-HRP with tyramide signal amplification. Sections were viewed on an upright fluorescent microscope equipped with a digital camera (Olympus).

### Real-time quantitative PCR

Long bones from hindlimbs of 7-day-old mice were stripped of soft tissue, cut into small pieces and homogenized in Trizol reagent (Sigma). RNA was extracted from homogenates and reverse transcribed into cDNA using a Superscript III kit (Invitrogen). Relative gene expression was determined by qPCR using established osteoblast and chondrocyte differentiation Taqman primer/probe sets in a 7500 Fast PCR machine (Applied Biosystems). The *GAPDH* gene was used as an internal control for all samples. Fold change in marker expression in SHP-2-deleted bones compared with littermate control bones was calculated as described (Schmittgen and Livak, 2008; Oliver et al., 2012). Experiments were repeated eight times and for each marker a mean fold change in expression and standard error was calculated.

### Statistical analysis

Statistical significance in quantitative PCR experiments was determined using a one-sample Student’s *t*-test.

## Supplementary Material

Supplementary Material
