# Supplementary material for: Deletion of SHP-2 in mesenchymal stem cells causes growth retardation, limb and chest deformity, and calvarial defects in mice
Source: Dis Model Mech. 2013 Sep 25;6(6):1448–58. doi: 10.1242/dmm.012849 (PMC3820267; doi:10.1242/dmm.012849)
Supplement: Supplementary Material [file supp_6_6_1448__index.html]

Deletion of SHP-2 in mesenchymal stem cells causes growth retardation, limb and chest deformity, and calvarial defects in mice — Deletion of SHP-2 in mesenchymal stem cells causes growth retardation, limb and chest deformity, and calvarial defects in mice — Supplementary Material 

# Deletion of SHP-2 in mesenchymal stem cells causes growth retardation, limb and chest deformity, and calvarial defects in mice

## DMM012849 Supplementary Material

**Files in this Data Supplement:**

- **Supplementary Material PDF**
