# Supplementary material for: Deletion of SHP-2 in mesenchymal stem cells causes growth retardation, limb and chest deformity, and calvarial defects in mice
Source: Dis Model Mech. 2013 Sep 25;6(6):1448–58. doi: 10.1242/dmm.012849 (PMC3820267; doi:10.1242/dmm.012849)
Supplement: Supplementary Material [file supp_012849_DMM012849.pdf]

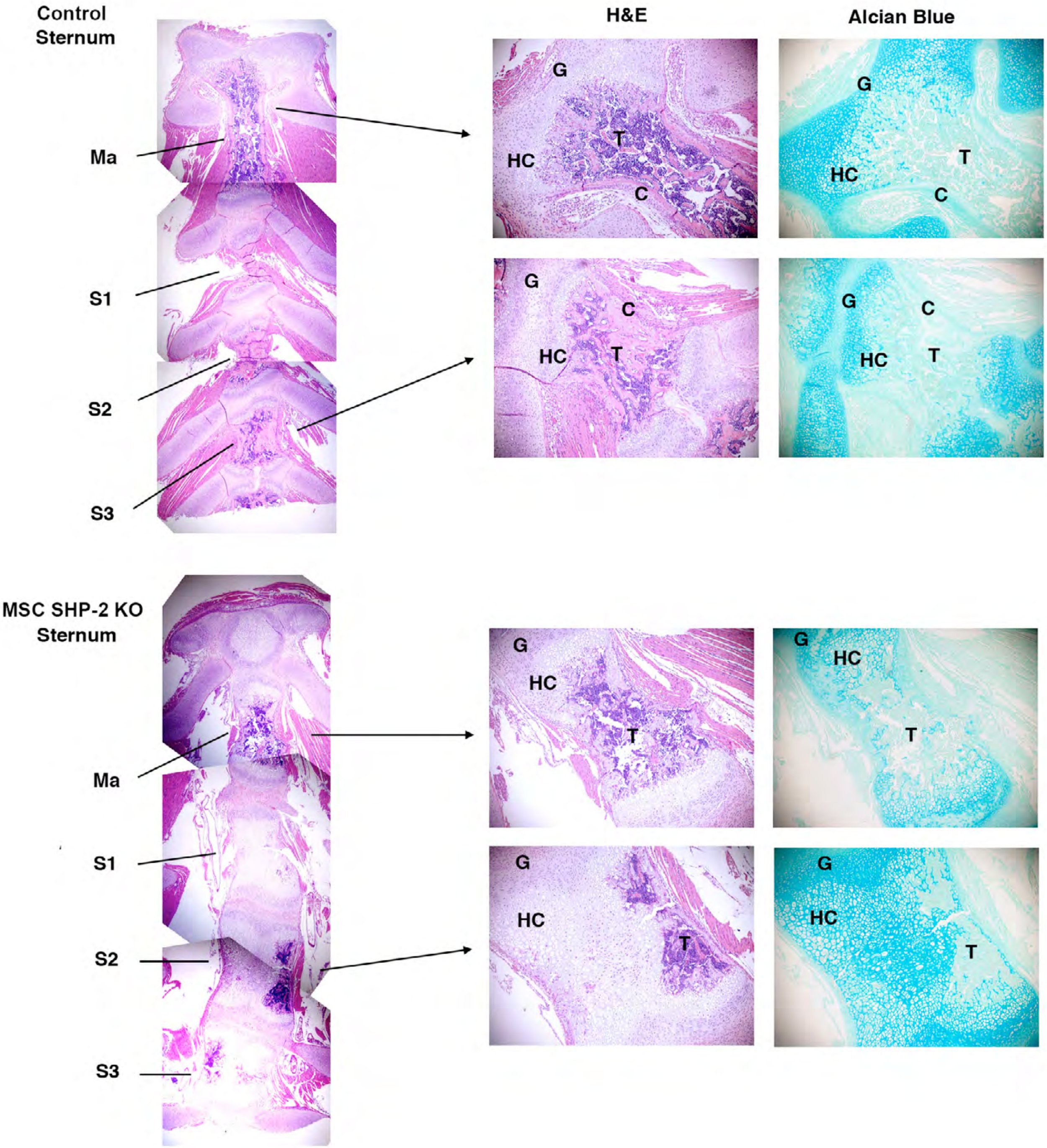

Fig. S1. Histology of MSC SHP-2 KO sternae. At left are shown composites of low power images of H&E stained sections of sternums of MSC SHP-2 KO and control littermate mice at 12 days of age (40X). At right are shown higher power images of the indicated select regions stained with H&E and of adjacent serial sections stained with alcian blue (100X). Note the absence of cortical bone and paucity of trabecular bone in MSC SHP-2 KO mice, particularly in S1-S3 sternebrae that comprise mostly of hypertrophic chondrocytes. Ma, manubrium; S1-S4, sternebrae 1-3; G, growth plate; HC, hypertrophic chondrocyte region; C, cortical bone; T, trabecular bone.

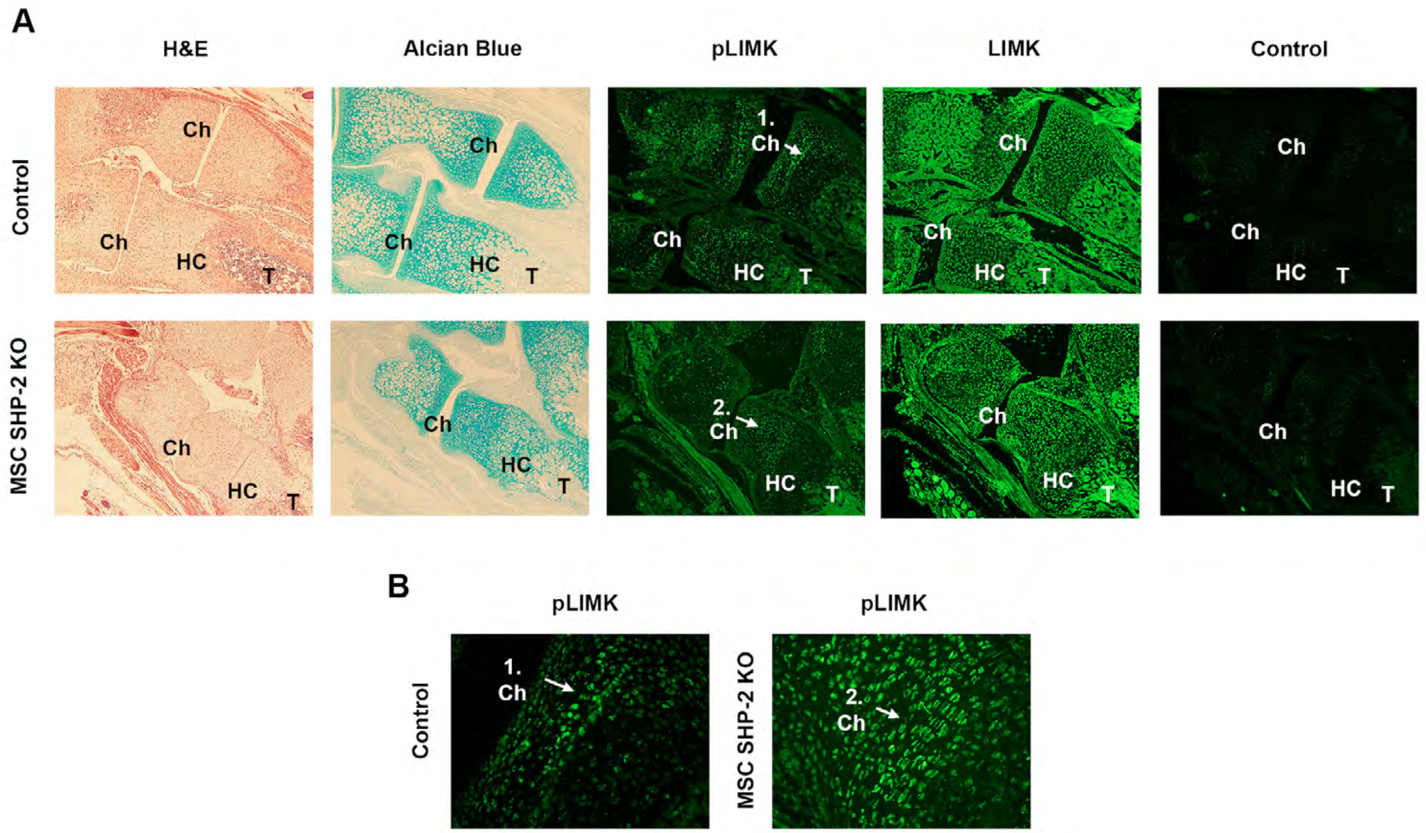

Fig. S2. Activation of LIMK1 in bone forming cells of MSC SHP-2 KO mice. (A) Serial sections of left hindlimbs of MSC SHP-2 KO and control littermate mice at 12 days of age were stained with H&E, alcian blue, anti-phospho-LIMK1 (pLIMK) or total LIMK1 antibodies followed by secondary reagents (green) or with secondary detection reagents alone (control). All images, 100X. (B) Shown are higher power images of the numbered regions (1 and 2) from (A) (400X). G, growth plate; T, trabecular bone; Ch, chondrocytes; HC, hypertrophic chondrocytes. Note pLIMK1 staining in chondrocytes of both types of mice.
